# Educational attainment, gender and health inequalities among older adults in Catalonia (Spain)

**DOI:** 10.1186/s12939-016-0414-9

**Published:** 2016-08-04

**Authors:** Aïda Solé-Auró, Manuela Alcañiz

**Affiliations:** 1 Department of Political and Social Sciences, Universitat Pompeu Fabra, C/ Ramon Trias Fargas, 25-27, 08005 Barcelona, Spain; 2 Riskcenter, Department of Econometrics, Statistics and Applied Economy, University of Barcelona, Av. Diagonal 690, 08034 Barcelona, Spain

**Keywords:** Health inequalities, Socioeconomic status, Education, Aging population, Logistic models, Spain

## Abstract

**Background:**

Health expectancies vary worldwide according to socioeconomic status (SES), with health disadvantages being evident among lower SES groups. Using educational attainment as a proxy of SES, we seek to identify trends in SES differentials in health by gender, with a particular focus on individuals with low educational attainment in the adult Catalan population (Spain) aged 55 or older.

**Methods:**

Using cross-sectional data for 1994 and 2010-2014 drawn from the Catalan Health Survey, we examined three health indicators to document social health inequalities: self-perceived health, functional limitations, and restrictions on activities of daily living (ADL). We applied logistic models for each indicator, controlling for sociodemographic characteristics, health coverage and health behaviours.

**Results:**

Among the less-educated, females presented a greater improvement in their self-perceived health over time than did their male counterparts, there being no significant variations among the medium/high educated. Regardless of education, males showed an increase in the prevalence of functional problems (as did the women, but the increase was not statistically significant). Both genders presented a higher prevalence of limitations when performing ADL in the second time period. The gender health gap was reduced slightly both for the low and the medium/high educated, expect in the case of ADL restrictions. Health and functioning differences by education level persisted, but showed significant signs of reduction.

**Conclusions:**

Less-educated females constitute the most disadvantaged group in terms of health and personal autonomy, though there are encouraging signs that the gap is closing both in terms of gender and level of education. Health policymakers need to devote particular attention to the aging population with low SES, especially to women. Public programmes promoting greater protection and equity, while fostering preventive and healthy practices, need to target the most underprivileged.

## Background

The remarkable gains made in longevity in Europe in recent years raise a number of questions about the corresponding impact on the health and quality of life of older adults and how these outcomes, in turn, are influenced by programmes designed to support the older population. Europeans are living longer and can expect to spend various decades in retirement beyond their working years; however, a significant part of their life expectancy at advanced ages is associated with disease and disability [1]. At the same time, it is well documented that socioeconomic status (SES) produces inequalities in health and its determinants. For instance, large variations in healthy life expectancy in relation to SES have been reported over time both across and within Europe [2, 3] and in the United States [4, 5]. These dissimilarities give rise to major health concerns and the need for policies that can cut health gaps by SES and promote active healthy aging in Europe for all, regardless of education and income [6–8]. Gender differentials also play a key role in promoting understanding of the complexity of inequality over time. Indeed, research has shown that women experience worse health but lower mortality than men [9, 10], and that these health differentials appear to be due to a combination of biological, behavioural, social, and economic differences as well as the interaction of these factors [11].

Reducing social health inequalities is a global concern. The aim of this study is to examine how recent trends in health by educational level and gender have interacted to affect health inequalities among the older population in Catalonia (Spain). We use the earliest as well as the latest data available to examine a period characterised by a marked expansion in education. We analyse the change in prevalence of three health measures, as well as gender health inequalities over time. Specifically, we examine whether improvements recorded in education have led to greater health inequalities, and whether these changes have had the greatest impact on the most vulnerable, namely, the low educated of both genders, but in particular women. Our attempts to focus on the most vulnerable groups are justified by the need to develop a greater understanding of how differentials arise and are maintained. Given the progress in education over roughly two decades (1994 to 2010-2014), we anticipate a reduction in social health inequalities. However, in the case of the gender health gap, we expect reductions across education levels, but anticipate that low-educated females will be the most disadvantaged.

### Determinants of successful aging

A number of components modify an individual’s life trajectory from birth to death. Rowe and Kahn [12, 13] introduced the term “successful aging”, a multidimensional concept involving the avoidance of disability and disease, the maintenance of high physical and cognitive function, and sustained engagement in social and productive activities. In general terms, the sizeable gains made in life expectancy can be attributed to successful public health policies, but they have also led to marked differences in the way older adults face the last stage of life. As Aureli and Baldazzi point out [14], an individual’s “registered age” and their “biological age” (their actual body age) no longer coincide. However, while in the developed economies of Northern and Central Europe (e.g. those of Sweden, Norway, Finland, Belgium, Denmark, the Netherlands, England and France), the marginal health returns of income are falling, especially in the higher income ranges [15, 16], economic resources continue to play a key role in health-related well-being at all ages, as they offer the possibility of converting income into goods and services that can promote healthy living [17].

Likewise, formal education is a fundamental factor in the way individuals face the end of their working lives and address aging, with the better educated having a healthier psychological function (greater mastery, efficacy, happiness) that sees them taking on a wider range of new commitments and opting for more innovative resources [18, 19], such as new cultural interests and virtual social networks.

In addition to income and education, other factors, including occupational status, owning a house, spiritual beliefs, lifestyle and material factors (absence of financial problems) have been identified as predictors of adjustment to aging [20, 21].

### The expansion of education in Spain

The evolution of Spain’s education system since the Spanish Civil War (1936-1939) has not had a uniform impact on successive generations of Spanish students [22]. Under Franco’s dictatorial regime, the State played what was primarily a subsidiary role in education, with schooling being dominated by the private sector, above all at the secondary level. However, the deterioration in living conditions of the Spanish population following the war made it impossible for the majority to gain access to these private institutions. This resulted in marked class-based inequalities in educational attainment. The Spanish population had to wait until 1970 – just five years before the death of Franco – to benefit from a comprehensive system of compulsory education until the age of 14. De la Fuente and Doménech [23] present a detailed description of the evolution in the educational attainment of the adult population in Spain between 1960 and 2010. They report a remarkable expansion in the average number of years of schooling in Spain, rising from 4.70 to 9.64 years between 1991 and 2010. During this sample period, illiteracy rates were low in most developed countries; yet, Spain, along with the other countries of Southern European, continued to present significant illiteracy rates. Over these four decades, the illiteracy rates almost disappeared, falling from 15.0 to 2.1 %.

### Socioeconomic differences in health by gender: the European context

The longevity advantage enjoyed by women contributes significantly to their health disadvantage [9]: “men die, women suffer” succinctly captures this gender health-survival paradox [24]. But whereas a considerable body of literature identifies a strong link between education and health in general [25, 26], it remains unclear as to whether low-educated women are particularly vulnerable and whether a low SES has a similar impact on the health of the genders.

Evidence from the Nordic countries shows that a high SES is closely linked to key healthy behaviours that boost life expectancy [27]. However, in Central and Southern Europe, lifestyles and cultural questions seem to be more closely related to risk factors and, hence, to disease and disability than are socioeconomic variables [28]. In Spain, at the end of the twentieth century, an improvement was recorded in the self-perceived health of the population, but the lower educated reported higher rates of poor health over time, without any distinction by gender [29]. Similarly, Alcañiz et al. [30] found that behaviours such as smoking, alcohol misuse and a sedentary lifestyle, as well as limitations for performing ADL, were associated with a greater risk of functional dependence among the Spanish, with the risk being higher for lower educated individuals, particularly women.

In the case of chronic conditions, Crimmins, Kim, & Solé-Auró [31], in an analysis of gender health differences across Europe and the United States, reported that while such differences are similar in direction they are somewhat different in magnitude. When the SES dimension in health is added, researchers have found a higher prevalence of hypertension and a greater risk of cardiovascular disease among less-educated females [32]. Fabbro et al. [33] argue that socioeconomically disadvantaged adults are less likely to be reached by health information campaigns and so are more vulnerable to lifestyle diseases with age. Additionally, these adults have to contend with restricted material conditions (e.g. a lack of access to healthy foods and limited opportunities of recreational physical activity in open spaces) and face adverse psychosocial problems (e.g. psychological stress associated with despair and problems of maintaining effective communication with others). The combined effect is the potential to undermine the health of the socioeconomically disadvantaged resulting in more co-morbidities [34].

## Methods

### Data

We drew our data from the 1994 and 2010-2014 Catalan Health Surveys (ESCA). The ESCA [35] is the only source of health-related microdata for Catalonia (Spain), a Mediterranean region with more than 7.5 million inhabitants in 2014. The Department of Health in Catalonia is responsible for conducting this official survey, which contains a wide range of information on individuals’ health-related behaviour and state of health, as well as sociodemographic variables. The sample follows a stratified design, based on age, gender and geographical area. The random collection of data is performed using personal interviews.

The cross-sectional survey was first collected in 1994 and then continuously for the period 2010 to 2014 [36]. Note, however, that the data are not longitudinal, as the respondents were chosen independently in the two time periods. In the second time period we aggregated four-year data (corresponding to the last semester of 2010, 2011, 2012, 2013 and the first semester of 2014) to increase our sample size. As a result, our sample comprises 10,307 randomly selected, non-institutionalized Catalan residents aged 55 years and older. Of these, 4446 individuals were interviewed in 1994 and 5861 were surveyed between 2010 and 2014. Henceforth, we refer to this latter period as 2010-2014.

### Measures

#### Conceptual health framework

Health is neither readily defined nor operationalized given its multidimensional nature. Primarily, however, attempts have been made to define it in terms of morbidity, and of functional and subjective health [37]. These various health dimensions constitute the disablement process, a pathway from disease to disability and death [38]. This process is dependent in part on the individual’s resources (income, health coverage, etc.), environmental factors (physical, intellectual, social, behavioural, etc.), and contextual factors (social situations, resources available to individual, etc.). As such, different health transitions can be identified in the course of the process.

#### Indicators

We use three health indicators, based on the conceptual framework of the disablement process, to disentangle health and disability statuses and, thus, to document social differences in health: 1) Self-perceived health: we consider those reporting themselves to be in ‘bad’ or ‘very bad’ health, as opposed to those who report being in ‘excellent’, ‘very good’ or ‘good’ health; 2) Physical and sensory functional limitations: this indicator of functional limitations is based on a respondent giving a confirmatory answer (yes vs. no) to at least one of the following five items: (i) limitations in seeing; (ii) limitations in hearing; (iii) mobility problems, such as being unable to get out of the house without help from another person; (iv) walking problems, which may require the use of special equipment; and (v) other significant mobility limitations, such as difficulty walking up and down a flight of stairs, and standing without using special equipment; 3) Restrictions on activities of daily living: difficulty in or need of assistance for eating, washing, getting dressed or toileting. ADL limitations, a more severe indicator, are usually located towards the end of the disablement process.

A range of variables have typically been used as indirect indicators of socioeconomic status, including family income, education and occupational status [39]. Here, we use education, since it tends to be stable after early adulthood, and relatively easy to measure, given that respondents usually report their educational attainment truthfully [40]. Moreover, and importantly, there is less likely to be reverse causation between education and health at older ages than there is with other measures of SES, such as income, wealth or occupational status [17]. Using the International Standard Classification of Education, we consider two groups based on the level of education attained: 0-2 for those with primary and lower secondary education (low educated), and 3-6 for those with upper secondary/tertiary education (medium or high educated).

In the regression we include marital status as a dichotomous variable (married vs. unmarried); occupation (employed, unemployed or inactive); self-reported smoking behaviour with three categories (non-smoker, past or current smoker); and alcohol intake, differentiating between at-risk drinkers and moderate or non-drinkers according to the classification provided by the Spanish Society of Family and Community Medicine [41]. Sedentary lifestyle reports individuals with no regular physical activity vs. those that engage in some. Spain provides universal public health coverage that can be complemented voluntarily with private health insurance. Thus, we also include controls for double health coverage.

### Analyses

#### Prevalence

We examine descriptive data on the prevalence of poor self-perceived health, functional limitations (sensory plus mobility) and ADL difficulties in individuals aged 55-plus by gender. We standardized our samples so as to be able to make comparisons between the two periods [42] and so each population has the same age and gender structure as that of the overall Catalan population on 1 July 2013, the date of the last official population register. Standardization weights were computed to preserve the original sample sizes of each period, so that the standard errors of the estimators were correctly calculated. Thus, any differences in our indicators resulting from a different demographic structure between 1994 and the period 2010-2014 are eliminated.

#### Binary logit models

We use logistic regression in each time period to examine trends in SES differentials by gender for the three outcomes. Sampling weights provided by the ESCA are used in the analyses to correct for age and gender deviations between our samples and the Catalan population in both time periods. Model 1 examines the effect on having selected health and disability indicators for each outcome controlling for age, sex, being married, and level of education. In order to understand fully how gender differences in education mediate gender differences in the prevalence of poor health, functional limitations and ADL limitations, we added an interaction in a second model. Therefore, Model 2 includes an interaction between gender and educational attainment plus double health coverage, and controls for unhealthy behaviours such as smoking (current or past), excessive drinking and sedentary lifestyle. The Model 2 interactions between gender and level of education are presented graphically for ease of interpretation. The plots show the predicted probabilities by education level and time period showing the gap between genders, and reporting 95 % confidence intervals for pairwise comparisons. As suggested by Goldstein and Healy [43], intervals have lengths equal to 2*1.39*standard errors giving an average level of 5 % for the type I error probability when comparing group means. Analyses are conducted using Stata software, version 13 (StataCorp).

## Results

Table 1 presents the sample characteristics for adults aged 55-plus in 1994 and in the period 2010-2014 by gender and level of education. In keeping with the expansion experienced by education in Spain, in 1994 a very high share of males and females presented a low level of education (88.6 and 94.4 %, respectively); however, these shares had been reduced considerably by the beginning of the 2010s (66.8 and 76.6 %, respectively). In the case of marital status, there was a more than 4 % decrease in both genders in the proportion of married individuals among the low educated, with no significant variations over time for the medium/high educated. In the case of occupation, the percentages of low-educated employed males fell by 4.3 %, with a similar increase for those reported as being inactive. Women with low education presented a very different pattern: employment rose by 4.4 %, while inactivity fell by 6.8 %. There were no relevant changes over time for the middle/high educated.

**Table 1:** Sample characteristics and age-adjusted prevalence of health and disability indicators by gender and level of education in 1994 and 2010-2014. Individuals aged 55-plus

		Men				Women			
		Low educated		Medium and High educated		Low educated		Medium and High educated	
		Prev.	CI 95 %	Prev.	CI 95 %	Prev.	CI 95 %	Prev.	CI 95 %
1994	**TOTAL**	**88.6**	**(87.2; 90.0)**	**11.4**	**(10.0; 12.8)**	**94.4**	**(93.5; 95.3)**	**5.6**	**(4.7; 6.5)**
	Married	83.1	(81.3; 84.9)	85.0	(80.4; 89.6)	58.5	(56.5; 60.5)	55.2	(46.9; 63.5)
	Occupation								
	Employed	20.6	(18.7; 22.5)	35.4	(29.2; 41.6)	6.7	(5.7; 7.7)	31.4	(23.7; 39.1)
	Unemployed	4.6	(3.6; 5.6)	3.6	(1.2; 6.0)	1.1	(0.7; 1.5)	0.0	-
	Inactive	74.8	(72.8; 76.8)	61.0	(54.7; 67.3)	92.3	(91.2; 93.4)	68.6	(60.9; 76.3)
	Health behaviours								
	Current smoker	25.9	(23.8; 28.0)	29.4	(23.5; 35.3)	1.4	(0.9; 1.9)	5.5	(1.7; 9.3)
	Past Smoker	42.8	(40.5; 45.1)	41.5	(35.1; 47.9)	1.5	(1.0; 2.0)	10.5	(5.4; 15.6)
	Drinking	5.2	(4.2; 6.2)	3.3	(1.0; 5.6)	1.7	(1.2; 2.2)	4.7	(1.2; 8.2)
	Sedentary lifestyle	24.1	(22.1; 26.1)	35.0	(28.8; 41.2)	30.7	(28.8; 32.6)	21.4	(14.6; 28.2)
	Double health coverage	15.0	(13.3; 16.7)	45.5	(39.0; 52.0)	16.5	(15.0; 18.0)	38.4	(30.3; 46.5)
	Health and disability								
	Poor self-perceived health	43.9	(41.6; 46.2)	24.2	(18.6; 29.8)	56.3	(54.3; 58.3)	31.8	(24.0; 39.6)
	Functional limitations	26.9	(24.8; 29.0)	15.7	(11.0; 20.4)	37.8	(35.8; 39.8)	18.1	(11.7; 24.5)
	ADL limitations	3.9	(3.0; 4.8)	1.9	(0.1; 3.7)	4.9	(4.0; 5.8)	3.5	(0.4; 6.6)
	Sample size, N	1746	-	227	-	2335	-	138	-
		Men				Women			
		Low educated		Medium and High educated		Low educated		Medium and High educated	
		Prev.	CI 95 %	Prev.	CI 95 %	Prev.	CI 95 %	Prev.	CI 95 %
2010-2014	**TOTAL**	**66.8**	**(65.1; 68.5)**	**33.2**	**(31.5; 34.9)**	**76.6**	**(75.1; 78.1)**	**23.4**	**(21.9; 24.9)**
	Married	78.6	(76.7; 80.5)	79.2	(76.6; 81.8)	54.2	(52.2; 56.2)	62.0	(58.4; 65.6)
	Occupation								
	Employed	16.3	(14.6; 18.0)	37.6	(34.5; 40.7)	11.1	(9.8; 12.4)	34.0	(30.5; 37.5)
	Unemployed	4.9	(3.9; 5.9)	5.7	(4.2; 7.2)	3.4	(2.7; 4.1)	5.0	(3.4; 6.6)
	Inactive	78.8	(76.9; 80.7)	56.6	(53.4; 59.8)	85.5	(84.1; 86.9)	61.0	(57.4; 64.6)
	Health behaviours								
	Current smoker	19.8	(18.0; 21.6)	22.1	(19.4; 24.8)	6.1	(5.1; 7.1)	14.4	(11.8; 17.0)
	Past Smoker	43.6	(41.4; 45.8)	41.7	(38.5; 44.9)	6.8	(5.8; 7.8)	21.5	(18.5; 24.5)
	Drinking	3.9	(3.0; 4.8)	2.0	(1.1; 2.9)	0.7	(0.4; 1.0)	1.3	(0.5; 2.1)
	Sedentary lifestyle	29.8	(27.7; 31.9)	32.9	(29.9; 35.9)	33.3	(31.4; 35.2)	29.3	(25.9; 32.7)
	Double health coverage	14.6	(13.0; 16.2)	37.5	(34.4; 40.6)	14.0	(12.6; 15.4)	40.3	(36.7; 43.9)
	Health and disability								
	Poor self-perceived health	41.1	(38.9; 43.3)	27.2	(24.4; 30.0)	50.6	(48.6; 52.6)	31.1	(27.7; 34.5)
	Functional limitations	36.1	(33.9; 38.3)	21.3	(18.7; 23.9)	41.4	(39.4; 43.4)	22.0	(18.9; 25.1)
	ADL limitations	10.2	(8.8; 11.6)	4.8	(3.4; 6.2)	13.8	(12.4; 15.2)	4.7	(3.1; 6.3)
	Sample size. N	1873	-	941	-	2343	-	704	-

Source: ESCA 1994, ESCA 2010-2014. Note: *Prev.* Prevalence, *CI* Confidence interval

Health behaviours changed over time, with some notable differences by gender and education. The prevalence of smoking among low-educated males fell by 6.1 % between 1994 and the period 2010-2014, whereas among similarly educated females it increased by 4.7 %. The largest increase in the prevalence of current smoking was recorded among females with a medium/high education (8.9 %). At the same time, both low and middle/high educated women reported rises in the prevalence of past smoking behaviour (5.3 and 11.0 %, respectively), while these rates remained steady for men. As for physical activity, only low-educated males presented an increment in sedentary lifestyle (5.7 %), while the rest of the groups remained stable. The percentage of population with double healthcare coverage did not present any significant changes by gender or level of education, with high-educated men being the most protected group.

We also recorded some statistically significant variations in the prevalence of the three health indicators by education when comparing the data between 1994 and the period 2010-2014. The perception of poor health among low-educated females fell over time (5.7 %). However, they presented a slight increase in functional problems (3.6 %; almost significant), and an 8.9 % increase in the prevalence of ADL limitations. At the same time, low-educated males also presented an increased prevalence of functional impairment (9.2 %) and ADL limitations (6.3 %). There was no statistical evidence of variations over time for those in the medium/high education group.

Tables 2 and 3 show the odds ratios (OR) of the explanatory variables indicating the effect on presenting ‘bad’ self-perceived health, functional problems and ADL limitations in 1994 and in the period 2010-2014.

**Table 2:** Estimates for binary logit models. Odds ratios indicating effect on presenting certain health and disability indicators: 1994. Individuals aged 55-plus

Variables	Poor self-perceived health		Functional limitations		ADL limitations	
	M1	M2	M1	M2	M1	M2
Constant	0.22***	0.36***	0.00***	0.00***	0.00***	0.00***
	(0.11;0.42)	(0.18;0.76)	(0.00;0.00)	(0.00;0.01)	(0.00;0.00)	(0.00;0.00)
Sociodemographic characteristics						
Age	1.00	0.98***	1.07***	1.05***	1.10***	1.07***
	(0.99;1.00)	(0.97;0.99)	(1.05;1.08)	(1.04;1.06)	(1.07;1.14)	(1.03;1.11)
Female	1.52***	1.76**	1.50***	1.72	1.78*	1.94
	(1.33;1.74)	(1.03;3.00)	(1.28;1.76)	(0.90;3.30)	(0.98;3.24)	(0.12;31.42)
Married	1.06	1.12	0.91	0.99	1.32	1.45
	(0.91;1.24)	(0.96;1.32)	(0.76;1.07)	(0.83;1.18)	(0.71;2.46)	(0.79;2.67)
Occupation (ref. employed)						
Unemployed	1.68**	1.62**	1.45	1.36	-	-
	(1.119-2.517)	(1.076-2.445)	(0.806-2.617)	(0.743-2.494)	-	-
Inactive	2.27***	2.25***	1.94***	1.96***	1.40	1.36
	(1.84;2.79)	(1.81;2.79)	(1.42;2.61)	(1.44;2.66)	(0.30;6.51)	(0.29;6.38)
Education						
Low Educated	2.36***	2.45***	1.89***	1.80**	2.12	1.44
	(1.82;3.06)	(1.61;3.72)	(1.37;2.60)	(1.09;2.98)	(0.49;9.12)	(0.20;10.53)
Education*Sex						
Low Educated*Male		1.02		1.15		1.90
		(0.59;1.76)		(0.59;2.23)		(0.11;33.93)
Health behaviours						
Current smoker		0.98		1.04		0.34
		(0.77;1.25)		(0.77;1.40)		(0.08;1.58)
Past smoker		1.44***		1.07		0.55
		(1.16;1.78)		(0.83;1.39)		(0.24;1.30)
Drinking		0.64**		0.51**		-
		(0.44;0.95)		(0.29;0.88)		-
Sedentary lifestyle		2.56***		3.27***		9.84***
		(2.20;2.97)		(2.80;3.83)		(5.11;18.93)
Double health coverage		0.78***		0.81**		0.83
		(0.65;0.92)		(0.66;0.99)		(0.43;1.60)
Observations	4167	4167	4167	4167	3917	3917
Pseudo R2	0.04	0.07	0.09	0.13	0.09	0.19

Source: ESCA 1994. Note: M1: Model 1; M2: Model2; The extremes of corresponding 95 % confidence intervals are indicated in parentheses; Significance is shown as follows: *** *p* < 0.01. ** *p* < 0.05. * *p* < 0.10

**Table 3:** Estimates for binary logit models. Odds ratios indicating effect on having selected health and disability indicators: 2010-2014. Individuals aged 55-plus

Variables	Poor self-perceived health		Functional limitations		ADL limitations	
	M1	M2	M1	M2	M1	M2
Constant	0.10***	0.16***	0.00***	0.00***	0.00***	0.00***
	(0.06;0.16)	(0.09;0.27)	(0.00;0.00)	(0.00;0.00)	(0.00;0.00)	(0.00;0.00)
Sociodemographic characteristics						
Age	1.01***	1.00	1.07***	1.06***	1.09***	1.05***
	(1.01;1.02)	(0.99;1.01)	(1.064-1.082)	(1.05;1.07)	(1.07;1.11)	(1.04;1.07)
Female	1.38***	1.31***	1.25***	1.24	1.58***	1.03
	(1.23;1.56)	(1.04;1.65)	(1.092-1.431)	(0.95;1.63)	(1.21;2.06)	(0.59;1.80)
Married	0.97	1.02	0.86**	0.91	0.94	1.02
	(0.85;1.11)	(0.89;1.17)	(0.748-0.999)	(0.78;1.06)	(0.72;1.24)	(0.77;0.34)
Occupation (ref. employed)						
Unemployed	1.50***	1.57***	1.32	1.46	-	-
	(1.11;2.03)	(1.14;2.14)	(0.850-2.052)	(0.92;2.31)	-	-
Inactive	2.10***	2.22***	2.05***	2.27***	4.64***	5.50***
	(1.76-2.52)	(1.84;2.68)	(1.600-2.636)	(1.74;2.95)	(1.82;11.85)	(2.09;14.47)
Education						
Low Educated	1.58***	1.81***	1.22**	1.41***	0.93	1.10
	(1.38;1.80)	(1.48;2.21)	(1.041-1.420)	(1.12;1.78)	(0.68;1.26)	(0.70;1.73)
Education*Sex						
Low Educated*Male		0.83		0.91		0.66
		(0.64;1.08)		(0.67;1.24)		(0.36;1.24)
Health behaviours						
Current smoker		1.05		0.91		0.82
		(0.87;1.27)		(0.72;1.15)		(0.48;1.41)
Past smoker		1.26***		1.31***		1.01
		(1.08;1.46)		(1.10;1.55)		(0.70;1.45)
Drinking		0.99		1.46*		1.61
		(0.65;1.51)		(0.95;2.26)		(0.64;4.04)
Sedentary lifestyle		2.55***		3.61***		10.47***
		(2.25;2.89)		(3.14;4.14)		(7.41;14.78)
Double health coverage		0.79***		0.99		0.68**
		(0.68-0.92)		(0.83;1.17)		(0.47;0.97)
Observations	5365	5365	5360	5360	5106	5106
Pseudo R2	0.04	0.08	0.13	0.19	0.14	0.25

Source: ESCA2010-2014. Note: M1: Model 1; M2: Model2; The extremes of corresponding 95 % confidence intervals are indicated in parentheses; Significance is shown as follows: *** *p* < 0.01. ** *p* < 0.05. * *p* < 0.10

As shown in Model 1, in general, age and being female were positively associated with all poor health and disability variables in both 1994 and 2010-2014. Indeed, the greater the severity of the health indicator, the greater was the influence of age. Being female contributed to a significantly increased likelihood of having poor self-perceived health, functional limitations and ADL limitations, with OR over 1.25 for both periods. Compared with being employed, being unemployed or inactive was associated with a higher likelihood of ‘bad’ self-reported health (OR over 1.50), while functional limitations only affected the inactive to a higher degree (OR around 2).

Being low educated also significantly increased the probability of having poor self-perceived health and functional limitations in both periods (OR over 1.22), which makes elderly women in this group additionally vulnerable. However, these OR decreased between 1994 and 2010-2014, indicating a reduction in the health gap between the low and medium/high educated.

Model 2 adds health behaviour controls and interactions between gender and level of education. The significant effect of the isolated gender variable is mitigated, although it remains significant for self-perceived health, which points to interrelations between education and gender, as well as to the different lifestyle behaviours of men and women. Having smoked in the past and leading a sedentary lifestyle were positively associated with reports of poor health for both periods. Specifically, physical inactivity was strongly linked to all the health conditions included. Excessive drinking was associated with a lower likelihood of poor self-perceived health and functional limitations in 1994 and appeared not to be relevant in the second period. Finally, double health coverage was linked with a lower probability of reporting health problems, functional limitations in 1994 and ADL in the period 2010-2014 (ORs 0.68 and 0.81, respectively).

The effects of the regressors on the presence of ADL limitations (a more severe situation that usually manifests itself at more advanced ages) are harder to detect as the samples of respondents affected are smaller. In both periods being older and female were significant risk factors (Model 1). Inactivity for the second time period, and sedentary lifestyle for both periods were also linked with higher likelihoods of ADL limitations (Model 2). Importantly, no evidence was found that education had a protective effect against ADL limitations.

**Fig. 1:**
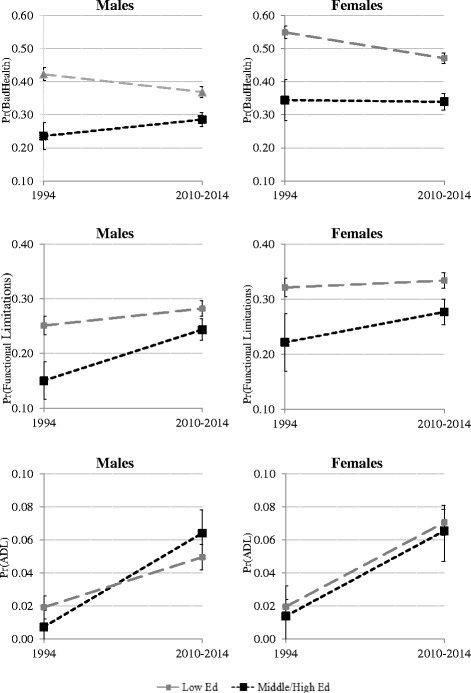
Predicted probabilities of poor self-perceived health, functional problems, and ADL limitations for males and females by education level and time period (CI: 95 %). Source: ESCA 1994, ESCA 2010-2014

Interestingly, the probability of presenting health problems varied both between and within males and females over these two decades. This is shown in greater detail in Fig. 1, which presents the results (in graph form) of the interactions between gender and education in 1994 and in the period 2010-2014. The predicted probabilities were calculated following our full model, and thus included controls for sociodemographic characteristics, health behaviours and double health coverage. In general, differences by gender were greater in 1994 than in the second period. Low-educated women were the most likely to perceive their health as being poor and to report functional limitations in both periods. However, the improvement in self-perceived health among the low educated was more marked for women than it was for men during this period, while the increase in the likelihood of suffering functional limitations was not so marked for women as it was for men. As a result, the gender gap between low-educated men and women was narrowed for self-perceived poor health and functional limitations between 1994 and the period 2010-2014.

As for those with a medium/high level of educational attainment, Fig. 1 shows an unfavourable evolution in terms of both their self-perceived health and their functioning capacity, the deterioration being more acute for men than for women. Thus, there is a slight reduction in the gender gap for this educational group. Although the changes are not statistically significant, it is worth noting that the gap between the low and the medium/high educated seems to have narrowed over time.

Finally, the level of educational attainment seems to have little impact on the presence of ADL limitations. While no clear pattern can be discerned by gender and/or education level, the predicted probability of suffering ADL limitations appeared to increase over the period for both men and women in both education groups.

## Discussion

Our findings reveal that the prevalence of functional and ADL limitations among those aged 55-plus in Catalonia (Spain) has increased since 1994 for all educational groups and for both genders. This finding is consistent with the expansion of morbidity reported by Solé-Auró and Alcañiz [1], and with the findings of Haro et al. [44] to the effect that the overall burden of years lived with disability increased by almost 26 % between 1990 and 2010 in Spain. An increased prevalence of disabling disorders has been linked to periods of economic crisis for the middle-aged and older adults [45], which may, in part, explain the trends observed here as the 2008 financial recession has hit Spain particularly hard.

Health inequalities have had an especially detrimental impact on females of all levels of educational attainment, with women not only facing more health issues than men but also over more years, as they live longer. A low level of educational attainment impacted men and women in a similar fashion with regard to poor levels of self-perceived health and functional impairment, and increased the vulnerable state of women. However, there are initial signs that the gap between males and females is being closed, both for the low and middle/high educated. In contrast, high levels of educational attainment appeared to be a protective factor against poor health and disability.

A number of encouraging findings were made with regard to these gender inequalities. Thus, the reduction over time in predicted probabilities of poor self-perceived health was more marked for women than it was for men, independently of whether they had a low or high level of educational attainment. In addition, the increment in the likelihood of functional limitations was less pronounced for women. These results seem to indicate that the gender health gap is gradually being closed, a process that might be attributed to the gender equality policies introduced some time ago by Spain’s social democratic government [46].

The effect of health behaviours differed between each SES group and between genders. The prevalence of smoking among females was greater in 2010-2014 than it had been two decades earlier in both educational groups, while the same trend was found for past smoking. In contrast, smoking decreased among low-educated men, but remained stable for the more educated. This fits with the temporal gap between gender cohorts identified by Bilal et al. [47], who point out that the female-to-male smoking ratio in Spain has risen over the last 50 years, with high-educated females being the first to show a reduction in their smoking prevalence. Sedentary lifestyle particularly affects low-educated men and its prevalence presents a worrying upward trend.

The sociodemographic and health profiles of individuals with public health coverage only and those with double coverage are known to differ; an outcome that is confirmed by our study. The literature reports that the Catalan population with double health coverage is composed predominantly of young adults from the more advantaged social classes, and that these individuals present fewer chronic conditions and less disability [48]. In contrast, low-educated individuals with only public coverage face restrictions that limit their universal protection (e.g. they have restricted access to specialist care) and face longer waiting times [49]. Moreover, in recent years their health has been threatened by the austerity measures adopted in Spain (and other European countries) as a consequence of the economic crisis [50]. Against a backdrop of economic difficulties and austerity policies, these inequalities need to be addressed not only by eliminating the cuts in Spain’s health and social service budgets, but also by achieving a more efficient and optimal management of resources. To avoid additional cuts in the universal coverage provided in Spain – a situation that threatens the health of the population [51], the implementation of more rational measures, including a reduction in the number of unnecessary diagnostic tests and in the amount of prescribed medicines, would serve to streamline costs without impairing the quality of the health care system.

There are a number of limitations in the analysis reported herein that might have affected our findings. Cohort effects, that is, the effects of being born at the same time, exposed to the same events in society, and influenced by the same demographic trends, might have impacted our findings. Moreover, capturing these effects when using cross-sectional data is not straightforward [52]. For example, the meaning attached to education may have changed in the time interval considered, as might have health returns on education. Likewise, the study of SES differentials in health would gain in clarity if longitudinal datasets could be exploited, but no panel data for Catalonia – or for Spain – are available. We have not considered the nursing home population; our results capture only non-institutionalized individuals. However, in 2006 the Catalan health authorities reported that the institutionalized population stood at about 0.7 % [53], so it is unlikely that the slight increase in the prevalence of poor health that their inclusion would have caused [54] is sufficient to modify our findings. Other explanatory variables (e.g. the body mass index) could have been included in our analysis, but the information available from the questionnaires – 1994 and 2010-2014 – is not always equivalent.

## Conclusions

Based on our findings, we believe that additional efforts need to be made to further reduce health inequalities by gender and education. The slight narrowing of the gap that has been detected both between genders and education levels should be strengthened by implementing social policies that foster equality and development. Specifically, the health care system must take into account the vulnerability of the least privileged and prioritize policies that can bridge the gap with the most favoured groups, rather than simply seek to prevent a greater deterioration in the health and autonomy statuses of the former. In this regard, policy interventions should be aimed at reducing health differentials in early life stages, where they can have a greater impact due to the accumulative effects of health behaviour over the lifespan.

If we look beyond the common protective and redistributive policies applied in Catalonia, an individual’s health and capacity to function are likely to be affected by various features of the national context (including the erosion of universal health coverage, income variation, and health-related practices) along with the differential exposure to risks associated with the level of education attained. Existing schemes designed to combat poverty result in low levels of deprivation, but much more can be done in the fight to reduce health inequalities. As such, policymakers need to begin implementing measures that promote equity so as to mitigate social disadvantages and to continue the struggle against poverty and deprivation [7].

At the same time, specific public programmes need to be designed to educate all sectors of the population in healthy habits. Avoidable malpractices, such as a sedentary lifestyle, poor diet, smoking and heavy drinking, may be just as much the result of an ignorance of the benefits of healthy habits [55] as being unable to afford healthy foods and goods. Healthy lifestyles – that is, engaging in sport and physical activity, giving up smoking, controlling drinking habits and eating a balanced, nutrient-rich diet – need to be promoted among the disadvantaged sectors of the population, along with ways of adopting them without necessarily incurring a great expense. Our findings should help policymakers develop a greater awareness of the relative benefits of boosting social and health protection and increasing prevention actions.

These health policy decisions should be based on the premise that the population presenting the lowest SES status – and most especially women – are more likely to suffer from poor health and disability as they age. To address these inequalities, national policies need to be re-evaluated so that the impact of individual characteristics on health [56, 57] may be modified in early life stages. In the first instance, individual health depends on the availability and quality of health care, prevention and protection throughout the national territory. Indeed, protective welfare state programmes may reduce health risks by compensating for material deprivation, and access to both education and medical assistance. Therefore, identifying and measuring the impact of social determinants on health is relevant, as it should be a way of determining just how social policies are designed and how generous they are [58]. As Haro et al. [44] suggest, shifting health care service provision from curative to preventive objectives should significantly reduce the burden of disease and disability.

While we recognize the limitations of our study, the analysis presented here for Catalans aged 55-plus clarifies that low educational attainment is associated with poor health and functional deterioration in both genders, adding to the general vulnerability suffered by women. The gender health gap has only been slightly narrowed, paving the way to future reductions. As for the value of our results, we would emphasise two aspects: first, the use we make of high-quality official data to disentangle health inequalities by educational level; and, second, the fact that we examine a social context – that of the population of Catalonia – where this relationship has hitherto been unexplored.

Health is not simply a multidimensional concept, but it is also a matter of social, economic, and political concern, with equity in health undoubtedly constituting one of the best indicators of social justice. Attempts to close the health gender gap, as well as efforts to eradicate disparities between low and high socioeconomic groups, offer great potential for achieving equity for all.

## Abbreviations

ADL, activities of daily living; ESCA, Catalan Health Survey; OR, odds ratio; SES, socioeconomic status
